# Chronic Liver Disease Presenting as Immune Hemolytic Anemia: The Challenges of Diagnosis in the Critically Ill in a Resource-Limited Health Care Setting

**DOI:** 10.7759/cureus.14880

**Published:** 2021-05-06

**Authors:** Kavita Gaur, Vandana Puri, Kiran Agarwal, Santosh Suman, Rajinder K Dhamija

**Affiliations:** 1 Department of Pathology, Lady Hardinge Medical College, Delhi, IND; 2 Department of Medicine, Lady Hardinge Medical College, Delhi, IND

**Keywords:** anemia, blood coagulation, hemolytic, fibrosis, liver

## Abstract

Immune hemolytic anemia is very rarely associated with chronic liver disease. Diagnosis is often complicated in critically ill patients, where an etiological diagnosis can be elusive, especially in routine health care settings.

A 48-year-old man presented with jaundice for three months. Ultrasonography showed features of chronic liver disease. Fibroscan showed increased parenchymal stiffness suggesting cirrhosis. Investigations revealed immune hemolytic anemia and thrombocytopenia. A percutaneous liver biopsy was not performed due to worsening thrombocytopenia. Isolated protein C deficiency and portal vein thrombosis were noted in subsequent testing. The patient eventually succumbed to illness.

Coagulopathy such as protein C and D-dimer elevation discovered in subsequent rounds of testing may be misleading in rapidly deteriorating patients, emphasizing the need for timely coagulation workup and imaging. Despite comprehensive testing, lack of liver biopsy, as seen herein, may hamper clinical management. Training residents in the skill of transjugular liver biopsy is necessary to manage critical patients at secondary health care facilities.

## Introduction

The role of the liver biopsy (LB) in clinical management is currently debatable. The invasiveness, associated risks, complications, and limitations of sampling have spurred the development of a number of non-invasive tests to assess liver dysfunction. Previous work has highlighted the pros and cons of the needle biopsy, with the current focus on improved imaging and the development of biomarkers to aid in diagnosis [1]. However, in this pursuit of devising “patient-friendly” non-invasive modalities, a lack of tissue diagnosis may be detrimental to patient care. Immune hemolytic anemia in association with chronic liver disease (CLD) is very rare. In addition, this report emphasizes the challenges of interpreting diagnostic tests, including coagulation assays in critical patients of liver disease. Systematic interpretation of hematological, biochemical, immunopathological, and fibroscan results may be useful in narrowing the differential diagnoses in CLD. Arriving at an exact etiological diagnosis in a scenario of clinical deterioration without biopsy tissue may be extremely difficult.

## Case presentation

A 48-year-old male presented with jaundice and abdominal distension for three months, with no other significant past or present history. On examination, the patient also had severe pallor. No signs of liver failure were noted. The liver and spleen were both palpably enlarged and firm, and ultrasound revealed coarse hepatic echotexture, suggesting chronic liver disease with ascites. Evidence of portal hypertension was not seen. Hemoglobin was 4.5 g/dl, platelet count was 30,000/µL, white blood cell count was 11000µL, and the differential leukocyte count showed a mild left shift.

**Figure 1:**
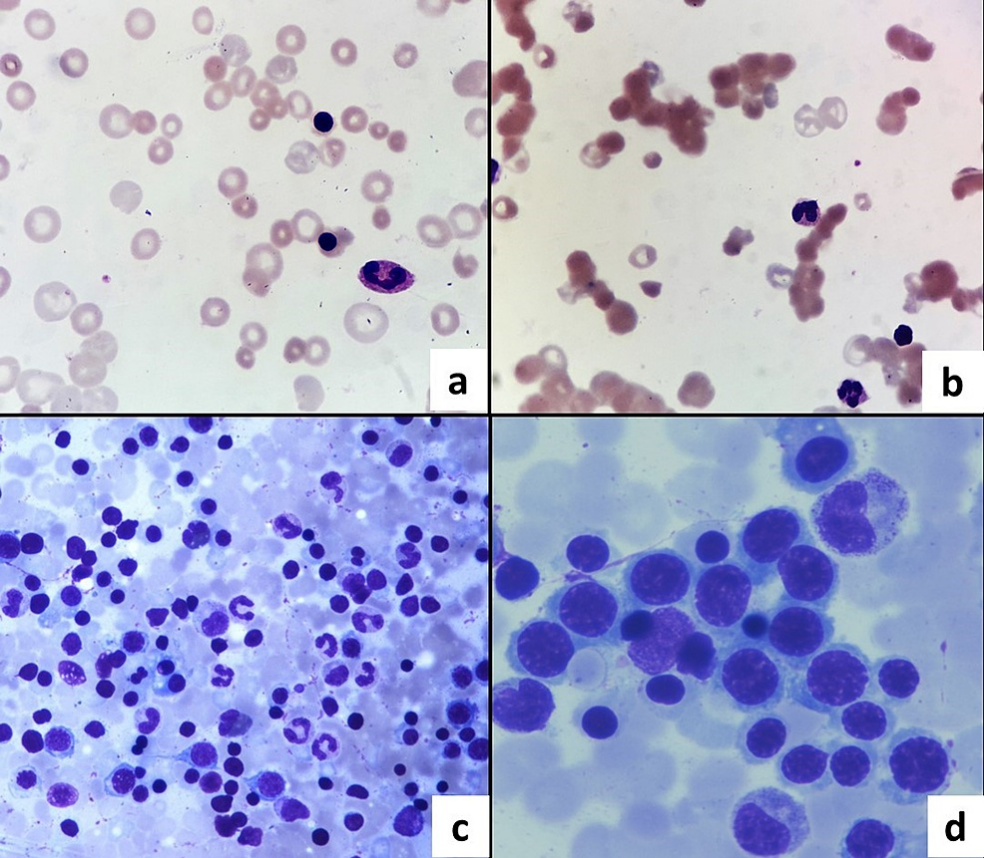
Peripheral smear photomicrograph (a-b) Peripheral smears show macrocytes, polychromatophilic cells, nucleated red blood cells (RBCs), microspherocytes, RBC agglutinates, left shift (Wright’s stain, 1000X) (c-d) Photomicrographs of bone marrow aspirate smears show erythroid hyperplasia without atypical cells (Giemsa, 100X, 400X).

Other investigations revealed unconjugated hyperbilirubinemia (3.4 mg/dl), elevated hepatic transaminases (alanine aminotransferase-147 IU/L, aspartate aminotransferase-297 IU/L), and hypoalbuminemia (2.6 g/dl). The initial peripheral smear revealed moderate anisopoikilocytosis, macrocytes, macroovalocytes, polychromatophilic cells, nucleated red blood cells (16/100 WBCs), few microspherocytes, and focal red cell agglutinates (Figure 1 (a-b)). The corrected reticulocyte count was 9.5%. No atypical cells were seen. Direct Coombs test was strongly positive (4+) and indirect Coombs test (3-cell and 11-cell panel) showed pan-agglutination. Serum B12 and folate levels and further workup for hemolytic anemia were unremarkable. The routine coagulation profile was insignificant, barring an elevated level of D-dimer (1074 ng/ml). Workups for viral infection, Wilson’s, and autoimmune disease were negative barring mild c-ANCA positivity (12 IU/ml). Serum alpha-fetoprotein was unremarkable. Ascitic fluid analysis revealed a total leukocyte count of 220 cells/cumm with 55% lymphocytes. Serum ascites albumin gradient (SAAG) was 1.9:1. Cytology showed reactive mesothelial cells and scattered lymphocytes. Adenosine deaminase levels were unremarkable, with acid-fast bacilli staining and genexpert test negative for mycobacterium tuberculosis. Bone marrow examination revealed normoblastic erythroid hyperplasia and no atypical cells (Figure 1 (c-d)).

A clinical impression of chronic liver disease with warm autoimmune hemolytic anemia (immunoglobulin G (IgG)) was suggested. LB was not performed on account of worsening thrombocytopenia. However, the fibroscan value was 27.0 KPa. The aspartate aminotransferase (AST)/platelet ratio index (APRI) score was 24.75. Fibrosis-4 (FIB-4) was 8.8.

The patient was administered intravenous ceftriaxone, which was stopped on the fifth day of admission, lactulose syrup, and folate supplementation. The patient was put on a regimen of prednisolone 50 mg once a day on the fifth day after cessation of antibiotic therapy. As the patient failed to respond to treatment, a hepatic doppler ultrasound was done, which revealed portal vein thrombosis. The total bilirubin had increased to 8.2 g/dl. Protein C level was 10 IU/dl (Reference: 65-135 IU/ dL) while protein S was unremarkable. The patient succumbed to illness three weeks after admission. Consent for postmortem liver biopsy/autopsy procedures was not given on account of religio-social factors.

## Discussion

Autoimmune hemolytic anemia (AIHA) with liver disease is a rare occurrence. Reports of cases of AIHA with autoimmune hepatitis, viral hepatitis, alcoholic liver disease, and non-alcoholic steatohepatitis have been documented [2-4]. Herein, investigations revealed immune hemolytic anemia with imaging suggesting chronic liver disease (CLD). In view of worsening thrombocytopenia and ascites, percutaneous LB was not performed. Standard guidelines recommend the performance of this procedure at a minimum platelet count of 50,000/µL [5]. Transjugular liver biopsy may be performed in such a scenario. The requirement of an interventional radiology set-up, the expense, and availability limit the performance of this technique. Here, it could not be performed, rendering etiological diagnosis difficult.

Viral, autoimmune, Wilson’s, and celiac disease serology was performed and was non-contributory. However, fibroscan results, AST/platelet ratio index (APRI), and fibrosis-4 (FIB-4) scores suggested advanced fibrosis. Hemolytic workup was also unremarkable, barring the Coomb’s test.

Doppler sonography done to rule out hepatic vasculopathy revealed portal vein thrombosis, raising the possibility of Budd Chiari syndrome. Further workup revealed D-dimer elevation and protein C decrease. These findings may reflect end-stage liver disease (ESLD) rather than a primary coagulopathy [6-7]. An elevated D-dimer correlates with the severity of liver disease and reflects an enhanced fibrinolytic state secondary to the reduced clearance of tissue plasminogen activator [7]. Coagulation tests must be interpreted with caution and complete clinicopathological correlation in the critically ill. Some authors opine a time-lapse of six months after an acute thrombotic event for accurate interpretation [8]. Others have suggested an immediate analysis to be useful only if followed by repeat testing after three months [9].

Evan’s syndrome or AIHA with immune-mediated thrombocytopenia was excluded in view of the concomitant advanced liver disease, CLD being a known cause of thrombocytopenia [10]. Though C-ANCA was elevated, granulomatosis with polyangiitis was not considered due to the lack of renal/upper respiratory involvement. C-ANCA elevation has also been described in association with ESLD. AIHA with thrombocytopenia has, in addition, been documented with lymphoproliferative disorders and infections like tuberculosis, endemic to the Indian subcontinent. Evidence of either malignancy or infection was not obtained in our case. AIHA with thrombocytopenia is well-documented in the literature, though AIHA in association with liver disease is a rare occurrence. Isolated reports of cases of AIHA with autoimmune hepatitis, primary biliary cirrhosis, viral hepatitis, alcoholic liver disease, and non-alcoholic steatohepatitis have been documented [2-4,11]. AIHA with thrombocytopenia, C-ANCA, and D-dimer elevation with protein C decrease as seen in this case, however, is uncommon and represents a clinical scenario that warrants further studies.

Despite a plethora of serological, biochemical, hematological, and imaging tests being done, the definitive diagnosis remained elusive and the patient succumbed to illness. Lack of LB diagnosis was a critical missing part of a challenging clinical picture of AIHA with rapidly worsening CLD. This report highlights the difficulties in arriving at an etiological diagnosis in critical patients with liver disease.

Though the LB is fraught with sampling limitations, a tissue diagnosis may definitely be useful in clinching or excluding various clinical possibilities.

## Conclusions

Warm AIHA is very rarely associated with liver disease and must be worked up thoroughly. Despite the advent of non-invasive tests to diagnose CLD, liver biopsy remains indispensable to determine etiology. The lack of liver biopsy and histopathological inputs on the same, as we learned, was the biggest lacuna of this case. Though existing literature is replete with AIHA and thrombocytopenia, co-existing protein C deficiency, C-ANCA rise, and D-dimer elevation, as seen herein, represents an area for further research. Efforts must be made to make available throughout low-income countries the expertise to perform the transjugular liver biopsy, which is imperative for obtaining liver biopsy tissue from critical patients. It is also essential to interpret coagulation tests cautiously in end-stage liver disease.
